# Modulation of the Apurinic/Apyrimidinic Endonuclease Activity of Human APE1 and of Its Natural Polymorphic Variants by Base Excision Repair Proteins

**DOI:** 10.3390/ijms21197147

**Published:** 2020-09-28

**Authors:** Olga A. Kladova, Irina V. Alekseeva, Murat Saparbaev, Olga S. Fedorova, Nikita A. Kuznetsov

**Affiliations:** 1 Institute of Chemical Biology and Fundamental Medicine, Siberian Branch of Russian Academy of Sciences, Novosibirsk 630090, Russia; kladova@niboch.nsc.ru (O.A.K.); Irina.Alekseeva@niboch.nsc.ru (I.V.A.); 2 Groupe «Mechanisms of DNA Repair and Carcinogenesis», Equipe Labellisée LIGUE 2016, CNRS UMR9019, Université Paris-Saclay, Gustave Roussy Cancer Campus, CEDEX, F-94805 Villejuif, France; Murat.SAPARBAEV@gustaveroussy.fr; 3 Department of Natural Sciences, Novosibirsk State University, Novosibirsk 630090, Russia

**Keywords:** DNA repair, AP endonuclease, single-nucleotide polymorphism, protein–protein interaction, coordination of DNA repair process

## Abstract

Human apurinic/apyrimidinic endonuclease 1 (APE1) is known to be a critical player of the base excision repair (BER) pathway. In general, BER involves consecutive actions of DNA glycosylases, AP endonucleases, DNA polymerases, and DNA ligases. It is known that these proteins interact with APE1 either at upstream or downstream steps of BER. Therefore, we may propose that even a minor disturbance of protein–protein interactions on the DNA template reduces coordination and repair efficiency. Here, the ability of various human DNA repair enzymes (such as DNA glycosylases OGG1, UNG2, and AAG; DNA polymerase Polβ; or accessory proteins XRCC1 and PCNA) to influence the activity of wild-type (WT) APE1 and its seven natural polymorphic variants (R221C, N222H, R237A, G241R, M270T, R274Q, and P311S) was tested. Förster resonance energy transfer–based kinetic analysis of abasic site cleavage in a model DNA substrate was conducted to detect the effects of interacting proteins on the activity of WT APE1 and its single-nucleotide polymorphism (SNP) variants. The results revealed that WT APE1 activity was stimulated by almost all tested DNA repair proteins. For the SNP variants, the matters were more complicated. Analysis of two SNP variants, R237A and G241R, suggested that a positive charge in this area of the APE1 surface impairs the protein–protein interactions. In contrast, variant R221C (where the affected residue is located near the DNA-binding site) showed permanently lower activation relative to WT APE1, whereas neighboring SNP N222H did not cause a noticeable difference as compared to WT APE1. Buried substitution P311S had an inconsistent effect, whereas each substitution at the DNA-binding site, M270T and R274Q, resulted in the lowest stimulation by BER proteins. Protein–protein molecular docking was performed between repair proteins to identify amino acid residues involved in their interactions. The data uncovered differences in the effects of BER proteins on APE1, indicating an important role of protein–protein interactions in the coordination of the repair pathway.

## 1. Introduction

The base excision repair (BER) pathway is one of the major pathways counteracting genotoxic effects of nonbulky DNA lesions frequently arising in the cellular genome [1,2]. The BER pathway is a multistep process that can be reconstituted using a limited number of proteins including a damage-specific DNA glycosylase, apurinic/apyrimidinic (AP) endonuclease, DNA polymerase, and DNA ligase [3,4,5]. It is believed that in BER, accessory proteins such as X-ray repair cross-complimenting protein 1 (XRCC1) and proliferating cell nuclear antigen (PCNA) participate in structural coordination of the aforementioned enzymes and help to organize an efficient substrate channeling between different enzymatic activities [6,7,8,9,10,11,12,13]. The passing of the baton in BER should involve the specific recognition of a product–enzyme complex by the next enzyme in the pathway [14,15,16].

Numerous studies of the protein–protein interactions between BER enzymes have shown that AP endonuclease enhances the turnover of many DNA glycosylases. Kinetic characterization of human monofunctional and bifunctional DNA glycosylases has revealed that their product release is rate-limiting during the steady-state phase of the reaction [17,18,19]. These observations suggest that human AP endonuclease (APE1) promotes the dissociation of the DNA glycosylase–product complex, and this effect increases several turnover rates of a number of DNA glycosylases [18,20,21,22]. Two mechanisms have been proposed to explain the stimulation of DNA glycosylases by APE1: a passive mechanism, when an AP endonuclease cleaves a non-occupied, free AP site to prevent its rebinding by a DNA glycosylase [17,22,23,24], and an active mechanism that consists of direct displacement of the DNA glycosylase from the AP site either via specific protein–protein interactions [18] or through distortion of the duplex DNA structure to disrupt the DNA glycosylase–AP site DNA complex [25,26,27].

Direct protein–protein interactions between APE1, DNA polymerase β (Polβ), and accessory proteins poly(ADP-ribose) polymerase 1 (PARP1), XRCC1, and tyrosyl-DNA phosphodiesterase 1 (TDP1) have been analyzed by fluorescence-based approaches [28]. These data have revealed strong protein–protein binding affinities. The physical interaction between APE1 and Polβ has been detected by various approaches [29,30,31]. It has been reported that downstream enzymes Polβ and XRCC1 stimulate the endonuclease activity of APE1 [32].

**Figure 1:**
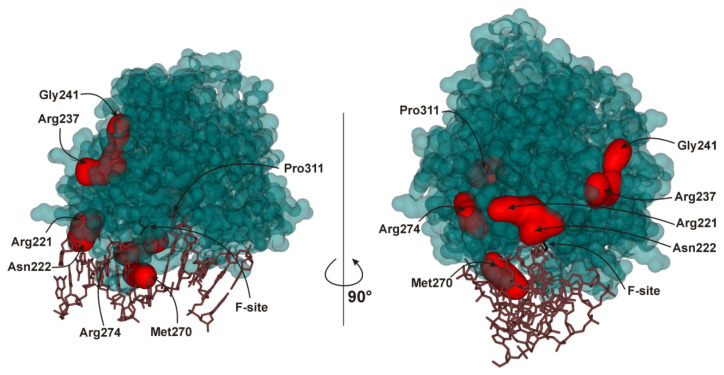
Spatial location of the amino acid residues affected by the SNPs of APE1 (PDB ID 1DE8).

Numerous studies of the direct interactions between proteins participating in BER are described in detail elsewhere [14,15,33,34,35,36]. The obtained data indicate that efficient repair of damaged DNA in the BER pathway proceeds via a tightly coordinated action of enzymes, where a preceding enzyme remains bound to its end-product DNA until it is displaced by the next enzyme in the reaction chain [36]. Nevertheless, the mechanisms of the substrate channeling and activity regulation within the BER pathway are still poorly understood in kinetic and structural terms. It seems that rather than being assembled into one stable multisubunit BER complex, these enzymes pass the repair intermediates among themselves by forming transient protein–protein complexes [37]. In this regard, it remains unknown how amino acid substitutions in the form of single-nucleotide polymorphisms (SNPs) in relevant proteins would affect the efficiency of the whole DNA repair pathway. Indeed, SNP-derived amino acid substitutions in BER enzymes are widespread in the human population. It should be noted that a decrease in a functional activity of individual BER enzymes and the disruption of either the coordination between them or coordination with accessory proteins can have many negative consequences. The roles of non-synonymous SNPs in the protein-coding region of BER genes in disease susceptibility have already been discussed in a number of publications [38,39,40,41,42,43,44,45,46,47,48,49,50,51,52]. Therefore, in the present study, we performed comparative analysis of the efficiency of DNA substrate channeling between various BER proteins and either wild-type (WT) APE1 or its natural polymorphic variants. We tested human DNA glycosylases responsible for the initiation of the BER pathway in the case of guanine oxidation (human 8-oxoguanine-DNA glycosylase; OGG1), cytosine deamination (human uracil-DNA glycosylase; UNG2), and purine alkylation (human alkyladenine-DNA glycosylase; AAG) as well as downstream enzyme Polβ and accessory proteins XRCC1 or PCNA to evaluate the influence of these proteins on the activity of APE1. Seven SNP variants of APE1 (R221C, N222H, R237A, G241R, M270T, R274Q, and P311S) were used for the kinetic analyses of the interaction with a DNA substrate containing a stable analog of an abasic nucleotide (F site) in the presence of various BER proteins. Spatial location of the affected amino acid residues was formally subdivided into several groups: surface amino acid residues Arg221, Asn222, Arg237, and Gly241; amino acid residues located near the DNA-binding site (Arg221, Asn222, Met270, and Arg274); and an inner residue: Pro311 (Figure 1).

These SNP variants of APE1 have been previously characterized in terms of DNA binding and catalytic activity toward an F site-containing DNA [53]. Even though these natural SNP variants of APE1, except for R237A, do not show [53] any dramatic changes in their catalytic activities, it was revealed here that a substitution of an amino acid residue located on the enzyme surface (Arg221, Asn222, Arg237, or Gly241) has a more significant influence on the protein–protein interactions with other BER enzymes than do substitutions of buried amino acid residues Met270, Arg274, and Pro311. Furthermore, in this study, molecular docking of WT APE1 to BER proteins of known X-ray structure was performed on the GRAMM-X web server [54] to identify a possible structural basis for the protein–protein interactions.

## 2. Results and Discussion

**Figure 2:**
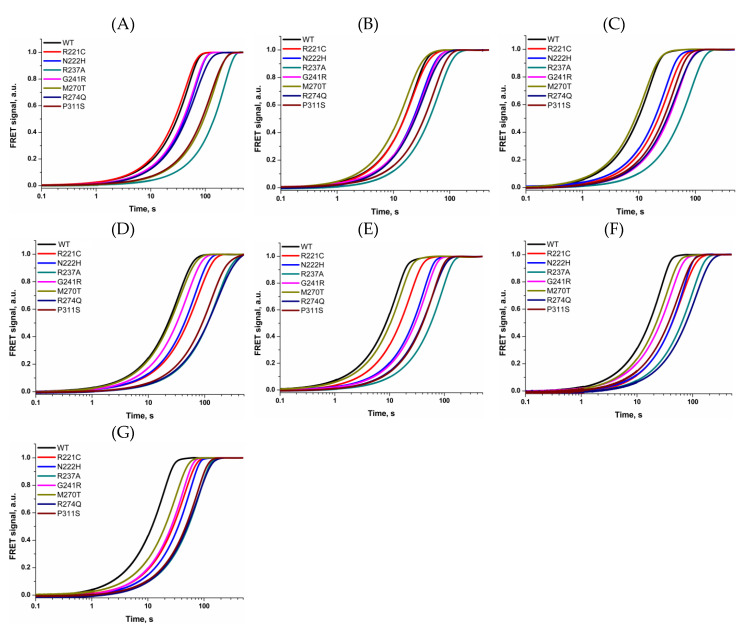
Interaction of WT APE1 or its SNP variants with the FRET-labeled substrate. The FRET signal changes characterize the activity of WT APE1 and SNP variants without any effectors (**A**) or in the presence of AAG (**B**), OGG1 (**C**), UNG2 (**D**), Polβ (**E**), PCNA (**F**), or XRCC1 (**G**).

In this study, we utilized a previously reported approach to determine the activity of several human BER enzymes, including AP endonuclease APE1 [55]. To determine the activity of WT APE1 and its SNP variants, we used a FRET-labeled DNA substrate (Table 1) containing an F site as a specific damaged nucleotide and dyes FAM and BHQ1 at the 5′ ends of the ODNs forming a duplex. WT APE1 and seven SNPs variants were analyzed to reveal the influence of AAG, OGG1, UNG2, Polβ, PCNA, and XRCC1 (i.e., effectors) on the efficiency of F site cleavage. This cleavage by APE1 and subsequent enzyme–product complex dissociation led to disconnection of the FAM fluorophore from the BHQ1 quencher, resulting in an increase in the FRET signal. As presented in Figure 2, the interaction of WT APE1 or its SNP variants with the FRET-F substrate caused an increase in the fluorescence signal up to time point 500 s, depending of the type of APE1 SNP variant and the presence of an effector protein.

**Figure 3:**
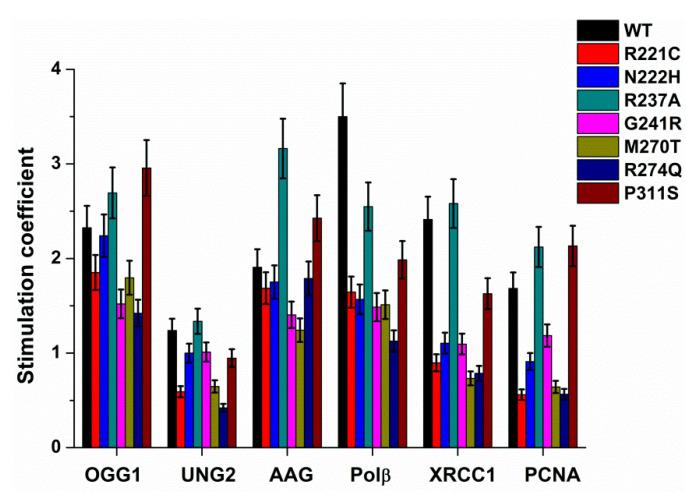
A comparison of stimulation coefficients *f* of BER proteins between APE1 WT and its SNP variants. The stimulation coefficient is computed according to Equation (2). [WT APE1 or SNP variant] = 10 nM, [FRET-F substrate] = 1.0 μM, [effector protein (AAG, OGG1, UNG2, Polβ, PCNA, or XRCC1)] = 1.0 μM.

The observed rate constants *k*_obs_ of the increase phase were calculated via Eq. 1 and are listed in Table 2. A comparison of the rate constants of F site cleavage between WT APE1 and its SNP variants without any effector proteins indicated that enzyme R237A possesses 5-fold lower activity, in good agreement with previously reported data [53,56]. These findings suggest that our FRET-based assay allows one to calculate observed rates that well describe the efficiency of the enzymatic reaction. The observed rate constants obtained in the presence and absence of an effector protein were utilized for computation of the stimulation coefficient (Figure 3), which characterizes the impact of the additional protein on the F site cleavage activity.

It was found that the activity of WT APE1 was stimulated by all the tested BER proteins. The greatest effect was observed in the presence of OGG1, Polβ, or XRCC1, when an increase in *k*_obs_ was more than 2-fold (Figure 3). The stimulation of the AP endonuclease activity of WT APE1 by Polβ has been demonstrated previously by product detection in a gel [29,30]; moreover, the stimulatory effect of XRCC1 on AP endonuclease activity was also reported very recently [32]. The stimulatory effects of AAG and PCNA were less than 2-fold, whereas UNG2 caused only insignificant enhancement of F site cleavage by WT APE1.

The comparison of the data obtained for the WT and SNP variants is suggestive of a possible role of each substituted amino acid residue (owing to an SNP) in the interaction with a given BER protein. A decrease or increase in the stimulatory effect as a result of an SNP respectively indicates either negative or positive involvement of the amino acid residue under study in the interaction. Under this assumption, it was found that the stimulatory effect of some BER proteins varies within a short range, most likely indicating the absence of direct contacts of the protein with the amino acid residues substituted due to the SNP. Indeed, in the case of UNG2, the range of stimulation coefficient *f* varies within a short range from 0.5 to 1.5 for all the tested APE1 variants. On the other hand, some SNP variants manifested a stable tendency of the stimulation effect regardless of which effector was applied. For instance, the substitution of Arg237, which is located on the surface of APE1, by Ala causes a significant increase in F site cleavage activity regardless of which effector is used. Moreover, the comparison of stimulation coefficients *f* between WT and R237A points to better activation of the latter by any effector except Polβ. Interesting to note, the data obtained for R237A indicate that this form has the lowest catalytic activity among the tested SNP variants, but, on the other hand, it is one of the most stimulated by other participants of the BER pathway. However, the activity of R237A even under stimulation by BER proteins was still less than the activity of the WT enzyme without any effectors (Table 2). The emergence of an additional Arg residue due to the G241R substitution on the enzyme surface also induces a noticeable decrease in the stimulation coefficient, suggesting that a positive charge in this area of APE1 (Figure 1) has a negative influence on the interaction with the other proteins. Substitution R221C, located on the enzyme surface near the DNA-binding site, results in permanently lower activation than that detected for WT APE1. By contrast, neighboring substitution N222H does not cause any significant difference from WT, indicating that Asn222 does not play an important role in these protein–protein interactions. These findings support the idea that positively charged surface Arg residues located in different regions of APE1 can participate in the protein–protein interactions but have opposite effects on the activity of APE1. Substitution P311S, buried in the APE1 globule, exerts opposite effects for some effector proteins. As compared with the WT enzyme, the P311S variant was more strongly stimulated by OGG1, AAG, and PCNA but less by UNG2, Polβ, and XRCC1. Both variant enzymes (M270T and R274Q) resulting from substitutions in the DNA-binding site are less sensitive than the other variants to the stimulation by BER proteins (for each effector, one of the two variants is always the least sensitive). This finding most likely means that the stimulatory effect of the BER proteins is restricted by the functional anomalies in a DNA-binding contact network that are induced by these substitutions.

**Figure 4:**
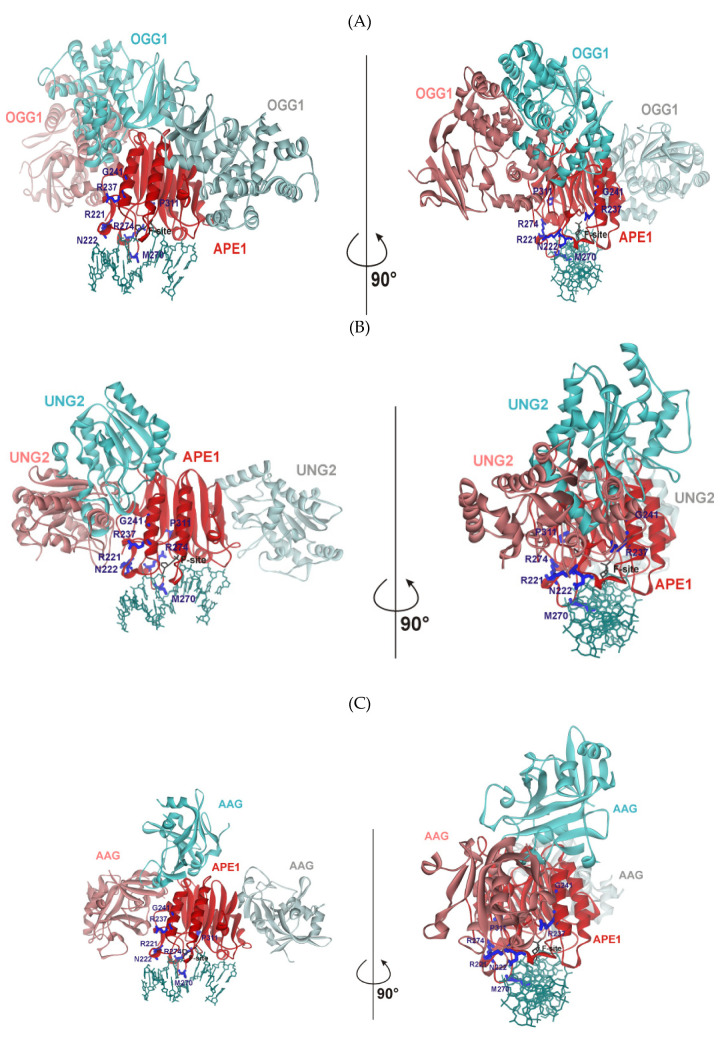

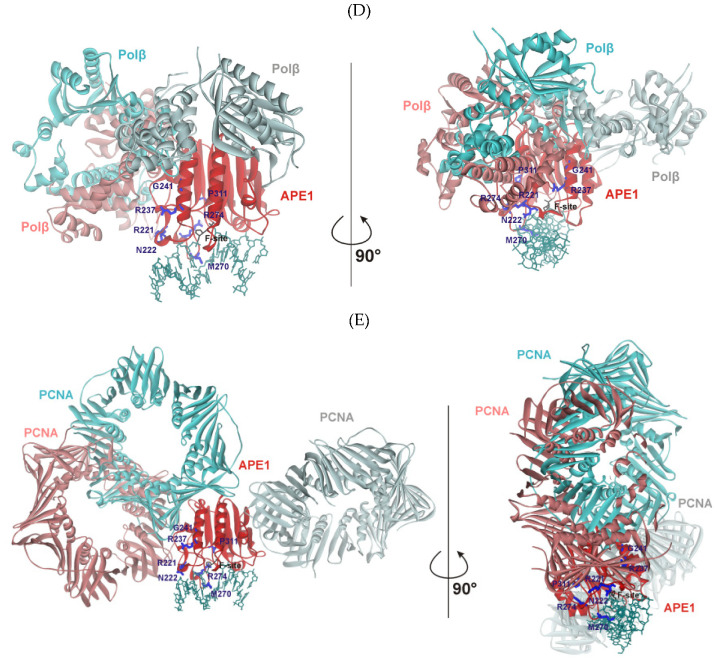
Protein–protein docked models of APE1 complexes with BER enzymes: OGG1 (**A**), AAG (**B**), UNG2 (**C**), Polβ (**D**), or PCNA (**E**). The PDB structures employed for the docking are 1DE8 for APE1, 1EBM for OGG1, 1F4R for AAG, 1EMH for UNG2, 4PHD for Polβ, and 6FCM for PCNA.

To identify the possible protein–protein interactions between WT APE1 and effector proteins, we docked all pairs of protein structures by means of the GRAMM-X web server [54], except for the APE1/XRCC1 pair, because a full-length structure of XRCC1 is unknown (Figure 4). The comparison of many docking models (see Supplementary Material) revealed the existence of cluster areas in which BER proteins are arranged more readily. In Figure 4, three versions of the spatial arrangement of an additional protein around the APE1 structure are depicted. It was found that each effector protein interacts with APE1 surfaces located close to the DNA-binding site. Moreover, the preferential position of binding of an additional protein to the APE1 surface turned out to be near the 3′ side of the damaged DNA strand. Of note, this cluster area located near the APE1 surface contains Arg221, Asn222, Arg237, and Gly241, implying potential participation of these amino acid residues in the protein–protein interactions.

**Figure 5:**
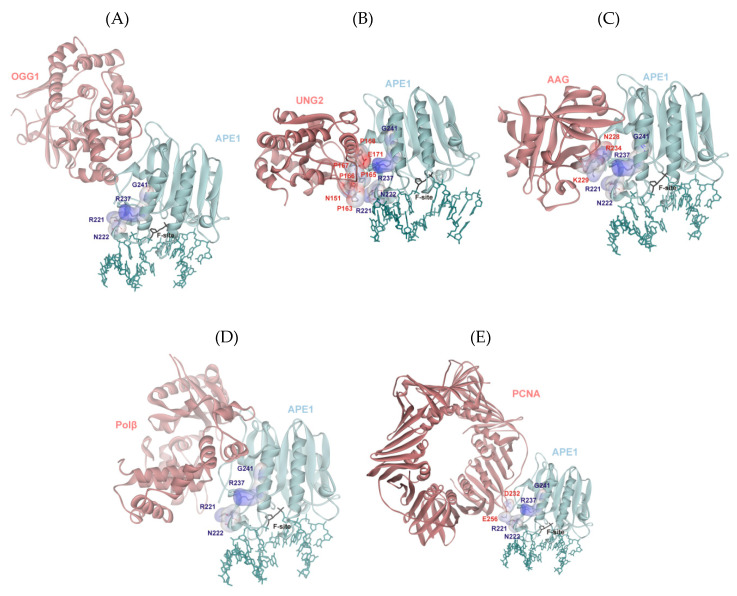
Possible amino acid interfaces participating in the protein–protein interactions around the area of a substituted amino acid residue in the SNP variants of APE1 as documented for OGG1 (**A**), AAG (**B**), UNG2 (**C**), Polβ (**D**), and PCNA (**E**). Surface amino acid residues of APE1 (Arg221, Asn222, Arg237, and Gly241) and amino acid residues of the effector proteins, if any, that were found near the SNP-induced substitutions in APE1 are illustrated in the transparent view of the electrostatic potential.

A close-up view of the protein from this cluster in the complex with APE1 suggested that UNG2 and AAG have their own surface amino acid residues that can potentially interact with Arg221 and/or Arg237 residues of APE1 (Figure 5). Even though residues Asn151 and Glu171 of UNG2 can stabilize the interaction with APE1, the proline-rich sequence from position 163 to 168 generates a significant positive charge on the UNG2 surface, which repulses the positively charged surface of APE1 (Figure 5A). A similar network of contacts involving Asn228, Lys229, and Arg234 was seen with AAG (Figure 5B). These observations are well consistent with the experimental data obtained on variants R221C and G241R, which revealed a correlation of the stimulation coefficient with the positive charge in this area on APE1 (Figure 3). Only stabilizing interactions of residues Asp232 and Glu256 with the APE1 surface were found in the model PCNA–APE1 complex (Figure 5C). On the other hand, the obtained model does not permit to identify any contacts of OGG1 and Polβ with the surface of APE1 near residues Arg221, Asn222, Arg237, and Gly241 (Figure 5D,E), meaning that the impact of these proteins on APE1 activity is mediated by another network of contacts with APE1.

## 3. Materials and Methods

### 3.1. Protein Expression and Purification

The WT APE1 enzyme and its SNP variants R221C, N222H, R237A, G241R, M270T, R274Q, and P311S were expressed and purified as described previously [53]. AAG and OGG1 were purified as described before [57,58].

The UNG2 enzyme was expressed in Rosetta 2 (DE3) *Escherichia coli* cells. The cells carrying the pET11a-UNG2 vector were grown in 1 L of Luria–Bertani (LB) medium supplemented with 100 μg/mL ampicillin at 37 °C until an optical density of 0.6 at 600 nm. UNG2 expression was induced by the addition of 0.2 mM isopropyl-β-d-thiogalactopyranoside (IPTG), and the cells were grown overnight at 37 °C. The cells were harvested by centrifugation (5000× *g*, 10 min) and then resuspended in a buffer (20 mM HEPES-KOH pH 7.8, 40 mM NaCl) followed by cell lysis under pressure using a SIM AMINCO French press. All purification procedures were carried out at 4 °C. Each homogenate was centrifuged at 40,000× *g* for 40 min, and the supernatant was passed through a column packed with 30 mL of Q-Sepharose resin (Cytiva, Marlborough, MA, USA) pre-equilibrated with the same buffer. The flow-through fractions containing UNG2 were pooled and loaded on a 1 mL HiTrap-Heparin™ column (Cytiva, Marlborough, MA, USA). Bound proteins were eluted with a 50–600 mM NaCl gradient. The purified UNG2 protein was supplemented with 50% of glycerol and then stored at −20 °C.

Proteins Polβ, PCNA, and XRCC1 were expressed in Rosetta 2 (DE3) *E. coli* cells. The cells carrying a pET28c expression vector were grown in 1 L of LB medium supplemented with 50 μg/mL kanamycin at 37 °C to an optical density of 0.6 at 600 nm. Then, the temperature was lowered to 20 °C, and transcription was induced by the addition of 0.2 mM IPTG. After induction, the cells were incubated for 16 h. The cells were harvested by centrifugation (5000× *g*, 10 min) and then resuspended in a buffer (20 mM HEPES-KOH pH 7.8, 40 mM NaCl) followed by cell lysis by means of the French press. All the purification procedures were carried out at 4 °C. Each homogenate was centrifuged at 40,000× *g* for 40 min, and the supernatant was passed through a column packed with 30 mL of Q-Sepharose resin (Cytiva, Marlborough, MA, USA) pre-equilibrated in 20 mM HEPES–KOH pH 7.8, 200 mM NaCl buffer. The flow-through fractions containing Polβ, PCNA, or XRCC1 proteins were pooled, supplemented with 20 mM imidazole, and loaded on a 1 mL HiTrap-Chelating™ column (Cytiva, Marlborough, MA, USA). Bound proteins were eluted with a linear 20 → 500 mM gradient of imidazole.

### 3.2. DNA Substrates

The sequences of the DNA substrates used in this work are shown in Table 1. Oligodeoxyribonucleotides (ODNs) were synthesized by the standard phosphoramidite method on an ASM-700 synthesizer (BIOSSET Ltd., Novosibirsk, Russia) using phosphoramidites purchased from Glen Research (Sterling, VA). The synthetic ODNs were purified by HPLC on an Agilent 1200 chromatograph (Agilent, Santa Clara, CA, USA) and a Zorbax SB-C18 column (5 mm), 4.6 × 150 mm, via a linear gradient of acetonitrile (0 to 50%) in the presence of 20 mM triethylammonium acetate, pH 7.0, for 30 min at a flow rate of 2 mL/min. The fractions containing ODNs were dried in a vacuum, dissolved in water, and precipitated with 2% LiClO_4_ in acetone. After a wash with pure acetone and drying, the ODN precipitates were dissolved in water and stored at −20 °C until experiments. Concentrations of the ODNs were determined by means of A_260_. Homogeneity of the purified ODNs was evaluated by denaturing 20% polyacrylamide gel electrophoresis with 8 M urea.

DNA duplexes were prepared by the annealing of modified and complementary strands at the 1:1 molar ratio in reaction buffer (50 mM Tris-HCl pH 7.5, 50 mM KCl, 1.0 mM EDTA, 1.0 mM DTT, 5.0 mM MgCl_2_, and 7% of glycerol).

### 3.3. Stopped-Flow Analysis

The real-time cleavage of the DNA duplex containing an F site was detected by the stopped-flow method with fluorescence detection as described elsewhere [55,59,60]. An SX.20 MV stopped-flow spectrometer (Applied Photophysics, Leatherhead, UK) fitted with a 150-W Xe arc lamp and a 2-mm path length optical cell was used. The dead time of the instrument is 1.0 ms. A FAM/BHQ1-labeled substrate was employed for the FRET analysis of the DNA cleavage reaction. The excitation wavelength (λ_ex_) of 494 nm was used for the FAM fluorescent dye, and the emission was recorded at λ_em_ > 515 nm (Schott filter OG-515). APE1 was placed in one of the instrument’s syringes and rapidly mixed with the substrate in another syringe. To reveal an influence of the tested BER proteins (effectors) on APE1 activity, the FRET-F substrate was preincubated with an effector protein (AAG, OGG1, UNG2, Polβ, PCNA, or XRCC1) to form the complex, and then this solution was mixed with the solution of APE1 supplemented by the same concentration of the effector protein. Final concentrations of the reactants in the reaction mixture were 10 nM WT APE1 or SNP variant, 1.0 μM FRET-F substrate, and 1.0 μM effector protein. The reaction was carried out at 37 °C in a buffer consisting of 50 mM Tris-HCl pH 7.5, 50 mM KCl, 1.0 mM EDTA, 1.0 mM DTT, 5.0 mM MgCl_2_, and 7% of glycerol. Typically, each trace shown is an average of three or more individual experiments.

The F site cleavage activity of WT APE1 and SNP variants was estimated by calculation of the observed rate constant *k*_obs_ using the following exponential equation:

F = F_0_ + F_1_ × exp(−*k*_obs_ × t) (1)

where F is the observed FRET signal, F_0_ is the background fluorescence, F_1_ is the fluorescence parameter, and *k*_obs_ is the observed rate constant.

The fitting procedure allows one to estimate the standard deviation, which is presented as an error value in Table 2. As can be seen in Table 2, the standard deviation typically has a very low value. On the other hand, all kinetic traces, which were fitted to Equation (1), are an average of three or more individual experiments. This procedure allows one to reduce the signal noise and to decrease effects of instrumental artefacts in the initial part of each individual trace. Fitting of these individual runs revealed approximately 5% difference in the values of observed rate constants, which allows us to estimate an experimental error of less than 5%.

The stimulation coefficient *f* was computed as a ratio of the observed rate constant in the presence of an effector protein, *k_obs_^P^*, to the rate constant characterizing the cleavage of the substrate by APE1 without any effectors: *k_obs_*^0^.

(2) $$f=\frac{k_{obs}^{p}}{k_{obs}^{0}}$$

Considering both sources of error: arising from the fitting procedure and arising from recording of kinetic curves by stopped-flow spectrometer, the total relative error of the stimulation coefficient was estimated as 10%.

### 3.4. GRAMM-X Web Server Docking

Using the GRAMM-X web server, the WT APE1 structure (Protein Data Bank [PDB] ID 1DE8, [61]) was docked with OGG1 (PDB ID 1EBM, [62]), AAG (PDB ID 1F4R, [63]), UNG2 (PDB ID 1EMH, [64]), Polβ (PDB ID 4PHD, [65]), or PCNA (PDB ID 6FCM, [66]). Protein–protein docking of APE1 and XRCC1 was not performed because of the absence of a full-length XRCC1 structure.

The number of alternative predictions to save in the final output file was 10. Among them, three models of each protein with APE1 were chosen as representatives of different clusters, which were found by a comparison of all the obtained models. The model with the closest location of an effector protein to the area of APE1 containing SNP-induced substitutions on the surface was employed to find the amino acid residues of the effector that could be important for the formation of contacts between the proteins.

## 4. Conclusions

Since APE1 performs a major function in the BER process, a missense mutation of this enzyme can increase the amount of DNA damage in the cell. Since BER process proceeds as a tightly coordinated sequence of enzymatic actions, it is easy to conclude that any disturbances in the interplay of BER proteins should decrease DNA repair efficiency. Moreover, such disturbances of repair can be associated with a natural SNP of a DNA repair protein gene. Indeed, multiple SNP variants of APE1 have been found to be distributed in the human population. Therefore, in this study, we performed kinetic analysis of an abasic site cleaving activity of WT APE1 and its SNP variants in the presence of other upstream (DNA glycosylase OGG1, AAG, or UNG2) or downstream (DNA polymerase β) BER enzymes as well as an accessory protein: XRCC1 or PCNA.

It was demonstrated that each of the following proteins, AAG, OGG1, Polβ, PCNA, and XRCC1, significantly stimulates the AP activity of WT APE1. The most obvious effect was seen with SNPs that change the positive charge of the enzyme surface near residues Arg237 and Gly241. The increase in the positive electrostatic potential on the surface decreases the stimulation coefficient of almost all the tested BER proteins. It is noteworthy that protein docking models revealed positive charges on the interacting surface of some enzymes, UNG2 and AAG; thus, these positive charges can cause repulsion of the protein molecules under study. Nonetheless, such a clear match between the stimulatory effect and surface charge was not observed in the models involving other effector proteins.

A substitution of another positively charged residue, Arg221, located near the DNA-binding site, suppresses the stimulatory effect. Moreover, a substitution of one of two active site amino acid residues, Met270 and Arg274, also significantly reduces the stimulation coefficient of any tested proteins. At the same time, neighboring Asn222 does not influence APE1 activity.

Thus, each of the tested BER enzymes exerts an influence on the F site cleavage activity of WT APE1 and its SNP variants. Comparative analysis of the efficiency of damaged DNA transfer from the complex with various BER proteins to wild-type APE1 or its natural polymorphic variants allowed us to improve the understanding of the mechanism of protein–protein coordination during the repair process. Nevertheless, elucidation of more precise molecular mechanisms of protein–protein interactions in the BER pathway requires additional mutational analysis.

## Figures and Tables

**Table 1:** The sequence of the DNA substrate used in the F site cleavage assay of WT APE1 and its SNP variants in the presence of BER proteins.

Name	Sequence
FRET-F substrate	5′-FAM–GCTCAFGTACAGAGCTG-3′ 3′-BHQ1–CGAGTGCATGTCTCGAC-5′

FAM is 6-carboxyfluorescein, BHQ1 is black hole quencher, FRET is Förster resonance energy transfer, and F (F site) is (2*R*,3*S*)-2-(hydroxymethyl)-3-hydroxytetrahydrofuran, a stable analog of an abasic nucleotide.

**Table 2:** Observed rate constants (*k*_obs_, s^−1^) of F site cleavage by WT APE1 or its SNP variants in the presence or absence of an effector protein.

Effector Protein	Variants of APE1							
	WT	R221C	N222H	R237A	G241R	M270T	R274Q	P311S
**No Effector**	0.021 ± 0.001	0.023 ± 0.001	0.015 ± 0.001	0.004 ± 0.001	0.019 ± 0.001	0.041 ± 0.001	0.014 ± 0.001	0.008 ± 0.001
**AAG**	0.040 ± 0.001	0.039 ± 0.002	0.026 ± 0.001	0.014 ± 0.001	0.027 ± 0.001	0.051 ± 0.001	0.025 ± 0.001	0.018 ± 0.001
**OGG1**	0.049 ± 0.001	0.043 ± 0.001	0.033 ± 0.001	0.011 ± 0.001	0.029 ± 0.001	0.073 ± 0.004	0.019 ± 0.009	0.022 ± 0.001
**UNG2**	0.026 ± 0.002	0.014 ± 0.001	0.015 ± 0.001	0.006 ± 0.001	0.019 ± 0.001	0.026 ± 0.001	0.006 ± 0.001	0.007 ± 0.001
**Polβ**	0.074 ± 0.001	0.038 ± 0.001	0.023 ± 0.001	0.011 ± 0.001	0.029 ± 0.001	0.062 ± 0.002	0.016 ± 0.001	0.015 ± 0.001
**PCNA**	0.035 ± 0.001	0.013 ± 0.002	0.014 ± 0.001	0.009 ± 0.002	0.023 ± 0.001	0.026 ± 0.001	0.008 ± 0.001	0.016 ± 0.001
**XRCC1**	0.051 ± 0.003	0.021 ± 0.001	0.016 ± 0.001	0.011 ± 0.001	0.021 ± 0.001	0.029 ± 0.001	0.011 ± 0.002	0.012 ± 0.001

## Supplementary Materials

The following are available online at https://www.mdpi.com/1422-0067/21/19/7147/s1.
